# Insulin-like growth factor-1 in articular cartilage repair for osteoarthritis treatment

**DOI:** 10.1186/s13075-021-02662-0

**Published:** 2021-10-30

**Authors:** Caining Wen, Limei Xu, Xiao Xu, Daping Wang, Yujie Liang, Li Duan

**Affiliations:** 1 Department of Orthopedics, Guangdong Provincial Research Center for Artificial Intelligence and Digital Orthopedic Technology, Shenzhen Second People’s Hospital, The First Affiliated Hospital of Shenzhen University, Shenzhen, 518035 China; 2 Department of Biomedical Engineering, Southern University of Science and Technology, Shenzhen, 518055 China; 3 Department of Child and Adolescent Psychiatry, Shenzhen Kangning Hospital, Shenzhen Mental Health Center, Shenzhen, 518003 China

**Keywords:** IGF-1, Cartilage repair, Chondrocytes, Osteoarthritis

## Abstract

Articular cartilage repair is a critical issue in osteoarthritis (OA) treatment. The insulin-like growth factor (IGF) signaling pathway has been implicated in articular cartilage repair. IGF-1 is a member of a family of growth factors that are structurally closely related to pro-insulin and can promote chondrocyte proliferation, enhance matrix production, and inhibit chondrocyte apoptosis. Here, we reviewed the role of IGF-1 in cartilage anabolism and catabolism. Moreover, we discussed the potential role of IGF-1 in OA treatment. Of note, we summarized the recent progress on IGF delivery systems. Optimization of IGF delivery systems will facilitate treatment application in cartilage repair and improve OA treatment efficacy.

## Introduction

Osteoarthritis (OA) is characterized by the progressive destruction of articular cartilage, which seriously restricts sports ability and impacts quality of life. The primary function of articular cartilage is to reduce the friction between joints and make joint movement smooth, soft, and painless [1]. Articular cartilage is mainly composed of chondrocytes and dense extracellular matrix (ECM) without blood vessels or innervation. Thus, chondrocyte metabolism in the adjacent cartilage is relatively low, and these cells cannot easily migrate to the damaged site [2]. Once a cartilage defect occurs, self-repair is not easy [3].

Currently, the typical treatment for the early stage of OA is anti-inflammatory drugs and hyaluronic acid injection. These conservative strategies can alleviate pain symptoms, but they cannot terminate the progression of cartilage deterioration and repair cartilage defects. Alternative therapies, including autologous chondrocyte implantation (ACI), matrix-induced ACI (MACI), and stem cell transplantation, are also available commercially or are in the clinical study phase. However, clinical efficacy of these treatments is not conclusive, as these treatments do not consistently regenerate hyaline cartilage. Finally, clinicians can use surgical methods such as joint replacement for the late-stage OA, which is a substantial economic and physical to patients and society. In addition to a series of risks during and after the joint replacement operation, total knee replacement surgery occurs in as many as 5% of cases, requiring revision surgery ten years after the operation [4]. Strategies to optimize OA treatment outcomes are urgently needed.

Growth factors and their signaling pathways have recently attracted much attention in cartilage repair for OA treatment. Insulin-like growth factor-1 (IGF-1), a member of a family of growth factors that are structurally closely related to pro-insulin, has shown profound effects on chondrocyte biological behavior and fundamentally regulates cartilage matrix metabolism during cartilage repair. Strategies for IGF delivery to chondrocytes and cartilage matrix are essential for its clinical application in OA treatment.

### IGF-1 in cartilage metabolism

#### IGF-1 promotes cartilage anabolism

Proteoglycan is an essential component of the ECM. An ex vivo study showed that IGF-1 in fetal bovine serum was responsible for maintaining articular cartilage proteoglycan synthesis. Thus, IGF has great potential to promote the regeneration of articular cartilage after injur y[5]. Another study has demonstrated that both serum and synovial fluid from rheumatoid arthritis (RA) could not stimulate chondrocyte proteoglycan synthesis once the IGF-1 function was blocked by a primary antibody [6]. Therefore, IGF-1 is the crucial factor in serum and synovial fluid that promotes cartilage matrix anabolism. Besides stimulating ECM production, IGF-1 can stimulate the proliferation and chondrogenic differentiation of mesenchymal stem cells (MSCs) [7].

In vivo experiments have similar results. In a rat fracture model, IGF-1 combined with TGF-ß could stimulate chondrocyte proliferation and cartilage formation at the early stage of day 5 [8]. IGF directly stimulates the proliferation of chondrocytes derived from rabbit ears, costal cartilage, articular joints, and growth plates [9, 10]. The presence of IGF-1 is crucial for maintaining cartilage integrity. When cartilage is damaged, MSCs derived from synovial fluid can partially move to the injured site and differentiate into chondrocytes to repair the defect, and IGF-1 induces chondrogenic differentiation [11–13]. An animal study in rats has confirmed that a continuous decrease in IGF-1 results in cartilage damage [14]. In addition, IGF-1 can stimulate the synthesis of type II collagen [15]. MSCs infected with the adenoviral (Ad) vector AdIGF-1 were fixed in fibrin glue and transferred to damaged rat cartilage. These cells could produce type II collagen and induce satisfactory repair results that were rich in protein aggregates [16]. In the sugar control group, the damaged area was empty or filled with fibrocartilage, composed of type I collagen. A further study [17] was performed to investigate the role of HIF-1α in cartilage regeneration using adeno-associated virus (AAV) and Ad vectors to overexpress IGF-1 in the knee joint of miniature pigs. Then, autologous periosteal cells combined with a scaffold overexpressing IGF-1 were implanted in the cartilage defect. The results showed that in the group that was administered periosteal cells without IGF-1 overexpression, a fibrous ECM was detected in the superficial layer of the defect, and there was little HIF-1α staining. Fibrocartilage matrix forms were identified in the deep layer and exhibited moderate HIF-1α expression. In contrast, periosteal cells with Ad/AAV-induced IGF-1 expression maintained a chondrocyte-like phenotype and produced a hyaline-like matrix with intense intracellular HIF-1α staining in the regenerated tissue area. This study indicates that IGF-1 can increase the expression of HIF-1α and that a simulated hypoxic environment is favorable for the regeneration of precursor cells in cartilage.

**Fig. 1:**
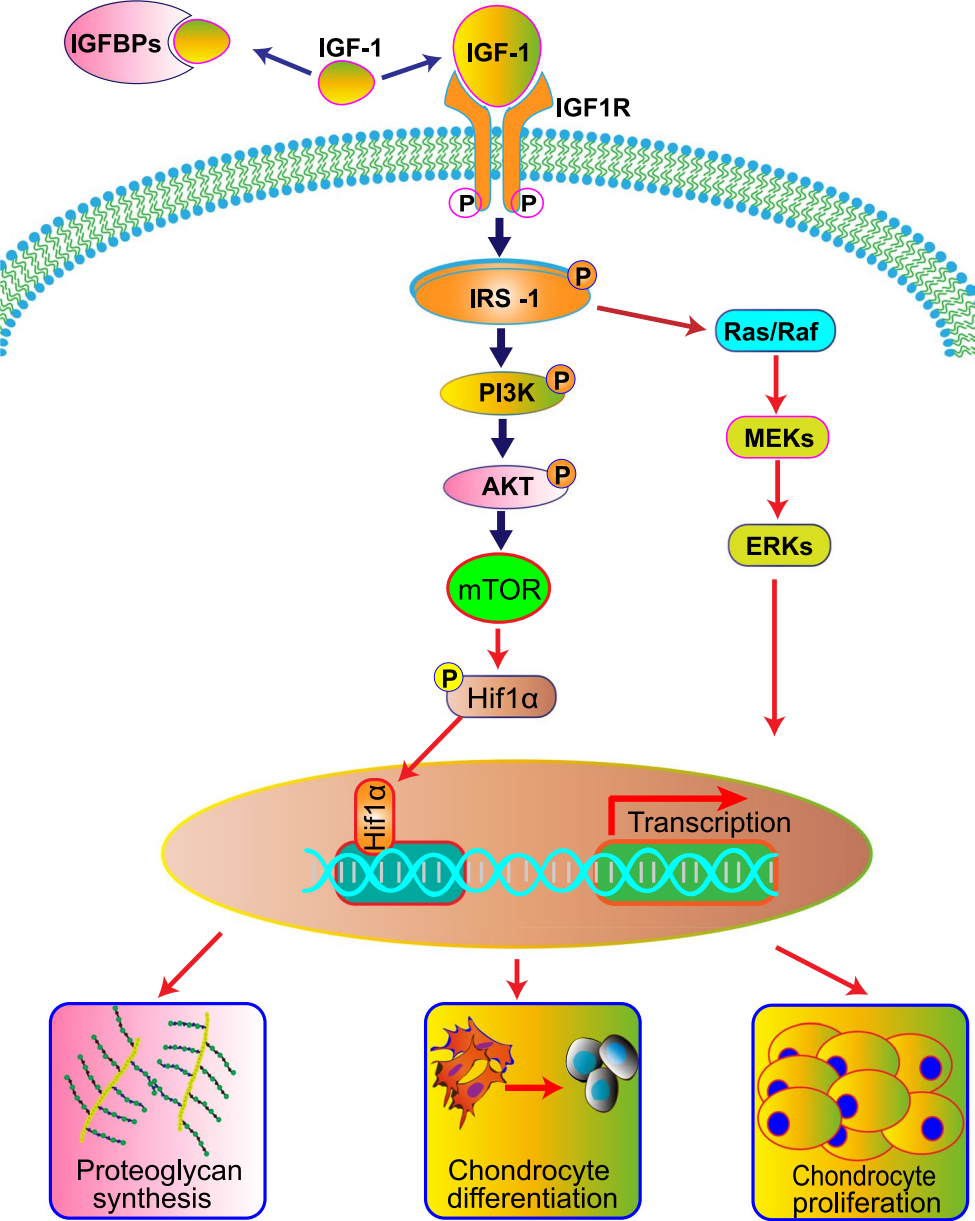
Schematic diagram of the IGF-I/PI3K/Akt and IGF-I/MAPK/ERK signaling pathways in articular cartilage metabolism. IGF-1 contributes to the chondrogenic differentiation of mesenchymal stem cells (MSCs). IGF-1promotes proteoglycan synthesis in chondrocytes by activating the PI3K pathway and stimulates chondrocyte proliferation via the PI3K and MEK/ERK pathways

IGF-1 promotes MSC chondrogenic differentiation and chondrocyte proliferation mainly through the activation of IGF-1R. The primary substrates recruited after IGF-1R activation are the insulin receptor-substrate (IRS) family members IRS-1 and IRS-2. Once IRS is phosphorylated, downstream signaling pathways are activated, including the phosphoinositide 3-kinase (PI3K) cascade and extracellular signal-regulated kinase (ERK). ERK is a member of the mitogen-activated protein kinase (MAPK) cascade [18]. IGF-1 activates PI3K through the IGF-1 tyrosine kinase receptor and activates the downstream target kinase Akt, thus activating mammalian target of rapamycin (mTOR). mTOR activation can induce chondrocyte proliferation and differentiatio n[11, 19]. The Ras and Raf/ MAPK/ERK (MEK) cascades are also activated during IGF-1-induced chondrocyte proliferation and differentiation. Figure 1 shows a schematic diagram of the IGF-1/PI3K/Akt and IGF-1/MAPK/ERK signaling pathways. An in-depth investigation suggested that IGF-1 stimulates human articular chondrocytes to synthesize proteoglycans by activating the PI3K/Akt/mTOR signaling pathway without activating the Ras/Raf/MEK/ERK pathway [18]. Additionally, IGF-1 may be associated with a low oxygen microenvironment since IGF-1 stimulates the production of hypoxia inducible factor-1α (HIF-1α) protein through the PI3K/mTOR and MEK/ERK pathways [20–22]. The essential role of HIF-1α has been recognized in joint cartilage formation and chondrocyte phenotype maintenance during development [23–25].

#### IGF-1 inhibits cartilage catabolism

When OA occurs, the ECM, especially type II collagen and aggrecan, is degraded. OA pathology is reversible in the early stage but irreversible at the late stage. It is well recognized that cartilage matrix degradation is stimulated by inflammatory factors, such as tumor necrosis factor-alpha (TNF-α) and interleukins (IL-1 and IL-7), and the production of matrix metalloproteinase-13 (MMP-13), which can degrade the major components of the ECM [26, 27].

Many studies have confirmed that IGF-1 can reduce the metabolism of the cartilage matrix. Amanda et al. found that adding IL-1 to cartilage explant cultures could degrade proteoglycans in the cartilage, and chondrocytes can take up IL-1 for further degradation. The addition of IGF-1 inhibits IL-1-induced matrix degradation [28]. IGF-1 inhibits IL-1β-induced NF-κB activation by inhibiting IκB-α kinase. NF-κB-regulated gene products are extensively involved in inflammation and cartilage degradation (COX-2, MMPs) and apoptosis (caspase-3). The inhibitory effects of IGF-1 on IL-1β-induced NF-κB activation are partially mediated by inhibiting the Src/PI3K/AKT pathway [29]. In addition, in rat endplate chondrocytes, the intervention of IGF-1 up-regulated the expression of MMP-13 through the ERK pathway, thereby inhibiting the decomposition of cartilage [30].

Chondrocyte apoptosis is a typical characteristic of OA. One of the IGF-1 functions is to protect chondrocytes from apoptosis [31, 32]. In a study by Higgins et al., IGF-1 was used to treat medial femoral condylar fractures in New Zealand white rabbits. Compared to the control group, the fibrin clot containing IGF-1 (25 μg/mL) for fracture repair inhibited chondrocyte apoptosis in vivo [33].

#### IGF-1 in OA progression

In addition to its effects on cartilage metabolism, IGF-1 affects the development of OA in other ways. The addition of IGF-1 led to the repair of cartilage and the repair of subchondral bone [34]. In another study, researchers explored the effects of different doses of IGF-1 on cartilage and subchondral bone repair. They found that high-dose IGF-1 was beneficial to cartilage formation, while low-dose IGF-1 was more conducive to subchondral bone reconstruction [35]. As mentioned above, IGF-1 can inhibit the production of IL-1 and metalloproteinase. Therefore, we can infer IGF-1 could alleviate synovitis with reduced inflammation. Furthermore, the ability of IGF-1 to reduce synovial inflammation has been proved in an equine model [36].

#### The risks of IGF-1 application

The relationship between IGF-1 and cellular senescence is still unclear and controversial. Some results suggested that IGF-1 could delay cartilage aging while some studies demonstrated that IGF-1 could accelerate the aging process of cartilage. In aging and dedifferentiated chondrocytes, researchers found that IGF-1 expression level was downregulated. In contrast, the aging factor was up-regulated, suggesting that reducing IGF-1 may result in the aging of chondrocytes [37]. Another study indicates that autocrine production of IGFs contributes to maintaining the survival of chondrocytes in vitro and in vivo [38]. However, the ability of IGF-1 to stimulate proteoglycan synthesis in chondrocyte decreases with the age of the donor increasing [39]. In another study on monkeys, the response of cartilage samples to IGF-1 significantly reduced with aging [40]. The mechanism may be related to the decrease of forkhead-box class O (FOXO) transcription factor expression in chondrocytes, thus weakening the cell's resistance to oxidative stress [41]. Then, oxidative stress inhibits IGF-1 to stimulate Akt phosphorylation and increases ERK phosphorylation, thereby inhibiting cartilage synthesis [39]. Controversial evidence suggested that IGF-1 could accelerate the aging of chondrocytes. Zhao et al. [42] found that IGF-1 promoted the senescence of rat articular chondrocytes by activating senescence-associated β-galactosidase (SA-β-gal) and up-regulating p53 and p21 expression levels. Thus, the interaction between IGF-1 and chondrocyte senescence need further investigation. Furthermore, whether IGF-1will lead to the excess production of cartilage, such as ectopic cartilage, is still a vacant field that we may make efforts.

#### IGF-1 in osteoarthritis treatment

Studies have shown that articular cartilage isolated from arthritic mice lacks a response to IGF-1 [43]. The reason may be that chondrocytes in OA produce excessive IGF receptor-binding protein, which blocks the activation of IGF receptors [44]. Thus, when using IGF-1 to treat OA, a simple injection of IGF-1 into the damaged site may not achieve a therapeutic effect.

**Fig. 2:**
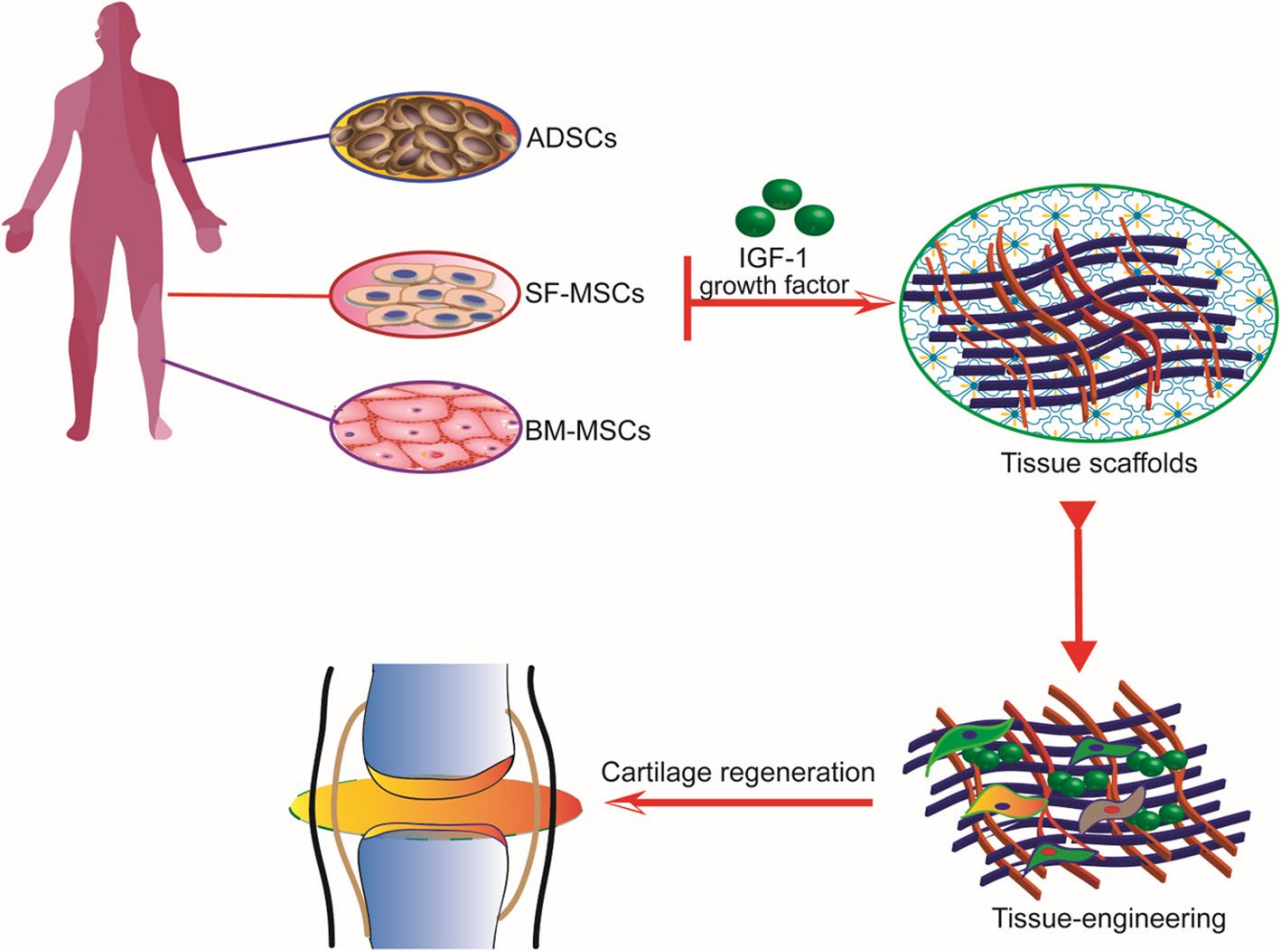
Tissue engineering-based IGF-1 delivery. MSCs, such as ADSCs, SF-MSCs, and BM-MSCs, are seeded on scaffolds. The addition of IGF-1 can induce chondrogenic differentiation of MSCs and promote cartilage tissue formation, cartilage matrix GAG accumulation, and type II collagen production, thus promoting cartilage regeneration and cartilage defect repair

Currently, most OA treatment with IGF-1 is based on tissue engineering. Cartilage tissue engineering involves seeding cells on a scaffold to support tissue growth and phenotypic maintenance by regulating growth factors and producing engineered cartilage with structures and functions equivalent to those of natural tissue [45, 46]. Tissue engineering combined with gene therapy is promising for articular cartilage repair. The transfection of articular chondrocytes with IGF-1 cDNA enhances the structural and functional properties of tissue-engineered cartilage based on polyglycolic acid scaffolds [47]. In another study [48], chondrocytes overexpressed IGF-1, and these genetically modified chondrocytes were cultured on a polyglycolic acid scaffold in vitro. The cartilage construct was implanted into the osteochondral defect of the knee joint in rabbits. After 28 weeks, the construct was analyzed. Compared to constructs without IGF-1 overexpression, IGF-1-overexpressing constructs significantly promoted osteochondral repair and reduced cartilage degeneration near the defect. IGF-1 delivery relies on tissue engineering methods, as shown in Fig. 2. Some studies on the application of IGF-1 via tissue engineering are shown in Table 1.

**Table 1:** Tissue engineering-based applications of IGF-1

Growth factors	Scaffolds	Cells	Effect	References
IGF-1/BMP-2	Fibrin glue	Mesenchymal cells isolated from rib perichondrium	Infecting mesenchymal stem cells with the adenoviral vectors AdBMP-2 and AdIGF-1 to induce transparent cartilage repair	[16]
IGF-1/TGF-β	Polyglycolic acid (PGA) scaffolds	Chondrocytes	The presence of IGF-1/TGF-β,or genetically modified chondrocytes overexpressing IGF-1 significantly improved osteochondral repair	[46, 48]
IGF-1	Type II collagen glycosaminoglycan (CG) scaffold	Adult articular chondrocytes	Continuous local overexpression of IGF-1 leads to enhanced cartilage formation	[49]
IGF-1/BMP-2	Bilayered oligo (poly(ethylene glycol) fumarate) (OPF) hydrogel composites	Mesenchymal stem cells	IGF-1 and BMP-2 can synergistically enhance the formation of subchondral bone, and the delivery of IGF-1 alone has a positive effect on the repair of osteochondral tissue	[50, 51]

Periosteum could be a very suitable tissue graft because it meets the three main tissue engineering requirements: seeding cells, a scaffold, and regulatory growth factors. Periosteum was transplanted into a defect and then developed into cartilage under the regulation of growth factors [52]. Matsumoto et al. [53] showed that cells in periosteal explants stimulated with IGF-1 (100 ng/mL) for two days differentiated into chondrocytes. Prolonged incubation (e.g., 6 weeks) of periosteal explants in serum-free medium led to type II collagen expression.

#### IGF-1 in clinical trials

The effect of IGF-1 on OA treatment has been extensively confirmed in vitro studies. However, the clinical evidence of IGF-1 in OA treatment is minimal. In prospective clinical trials (study number DRKS00000365 and UKF001822), by analyzing the growth factor content in the synovial fluid of patients with OA, they found the IGF-1 concentration in the joints with cartilage lesions was significantly increased compared to healthy samples [54]. The increase of local IGF-1 binding protein prevents IGF-1 from freely exerting its biological effects [55]. Therefore, IGF-1 delivery strategies for OA treatment should be optimized. In addition, IGF-1 based-target therapies are under consideration to avoid its side effects.

#### Strategies for IGF-1 delivery

IGF-1, as a small and highly diffusible protein, can be transported long distances to exert its biological effects [56]. However, as a molecule with close structural homology to insulin, IGF-1 elicits a variety of metabolic products in vitro and in vivo that are similar to insulin, which can cause edema primarily in the face and hands, mild weight gain, occasional dyspnea, bilateral jaw tenderness, arthralgias and myalgias, fatigue, tachycardia, flushing, orthostatic, and hypotension [57]. These long-term side effects and the risks of carcinogenesis [58] seriously compromise the systemic application of IGF-1. The biggest obstacle regarding local delivery is inflammation-induced IGF-1 degradation [59]. Therefore, considerable efforts have been made to improve IGF-1 delivery technologies.

#### Gene delivery

In vitro and in vivo studies have proven the efficacy of IGF-1 gene therapy in OA treatment [16, 60, 61]. Viral and nonviral vectors have been used for IGF-1 gene delivery. The advantages and disadvantages of both systems are described in Table 2.

**Table 2:** The pros and cons of different genetic methods for IGF-1 delivery

Vectors	Advantage	Disadvantage	References
Retroviral vector	Long-term expression of genes incorporated into host cells	Insertion mutations, difficult to terminate expression, premature senescence of cells	[1, 62]
Adenoviral vector	High transfection efficiency and high level of gene products	Severe immunogenicity	[63]
Adeno-associated virus vector	Excellent long-term gene transfer efficiency, no immune response, and a lack of toxicity and mutagenesis	Small target genes can be inserted	[64, 65]
Lentiviral vector	Larger cDNA can be introduced into target cells without causing immune responses	Safety unknown	[66]
Nonviral gene delivery	Improved safety	The long-term effect is not good	[1]

At present, viral vector-mediated gene transfer of IGF-1 has been widely documented. Kyla et al. [67] showed that chondrocytes from horses that were transduced with rAAV5-IGF-1 exhibited improved histological scores, including increased chondrocyte predominance and collagen type II production. Ad vectors are the most popular viral vectors and have high transfection efficiency. In one study [16], AdIGF-1-infected MSCs were fixed in fibrin glue and implanted in rat cartilage defects, which led to satisfactory cartilage repair (type II collagen matrix secretion and hyaline cartilage production). However, Ad vectors may trigger severe host reactions, including causing patient death during the first phase of gene therapy clinical trials [63]. Therefore, more substantial evidence is still needed to support the clinical application of Ad vectors.

Nonviral gene delivery refers to DNA passage through cell membranes using physical and chemical methods such as electroporation. Nonviral gene delivery systems are advantageous compared to virus transfection because these methods can avoid mutations caused by retroviral gene transfection and reduce the immune response. Moreover, the cargo is not inserted into the target cell genome. Biological safety guarantees the application of this delivery system in chronic disease treatment that requires long-term export of gene products [1]. A scaffold made up of type II collagen and glycosaminoglycan have been used as a nonviral gene delivery vector. The scaffold was soaked in an IGF-1 plasmid solution with a Lipofection reagent. Rapid release and sustained IGF-1 overexpression resulted in significant glycosaminoglycan accumulation and type II collagen production [49].

#### Protein delivery

In addition to gene delivery, various IGF-1 protein delivery systems have been developed. Tokunou et al. [68] designed and purified a new protein named heparin bound to IGF-1 (Xp-HB-IGF-1). The researchers found that Xp-HB-IGF-1 was selectively retained by cartilage explants and led to continuous chondrocyte proteoglycan biosynthesis. Julian C. Lui et al. [69] developed a cartilage-targeted single-chain human antibody fragment (CaAb) and fused this fragment to IGF-1. The CaAb-IGF-1 fusion protein had sustained cartilage binding ability and IGF-1 biological activity, promoting cartilage formation in model mice.

In addition, small cationic nanocarriers (< 15 nm) can overcome the biological barrier of joints by binding and penetrating anionic cartilage tissue. Studies have shown that the penetration speed of these nanocarriers was faster than the rate of carrier clearance by the joint space [70, 71]. IGF-1 was loaded into amine-terminated polyamide-amine dendrimer nanocarriers, and then different polyethylene glycol concentrations were used to control the surface charge to enhance local function [72]. The tissue uptake formula increased the residence time of therapeutic IGF-1 in the rat knee by ten times and showed improved cartilage repair. This study demonstrated that the nanocarrier could improve the pharmacokinetics and clinical efficacy of IGF-1 for OA treatment.

**Fig. 3:**
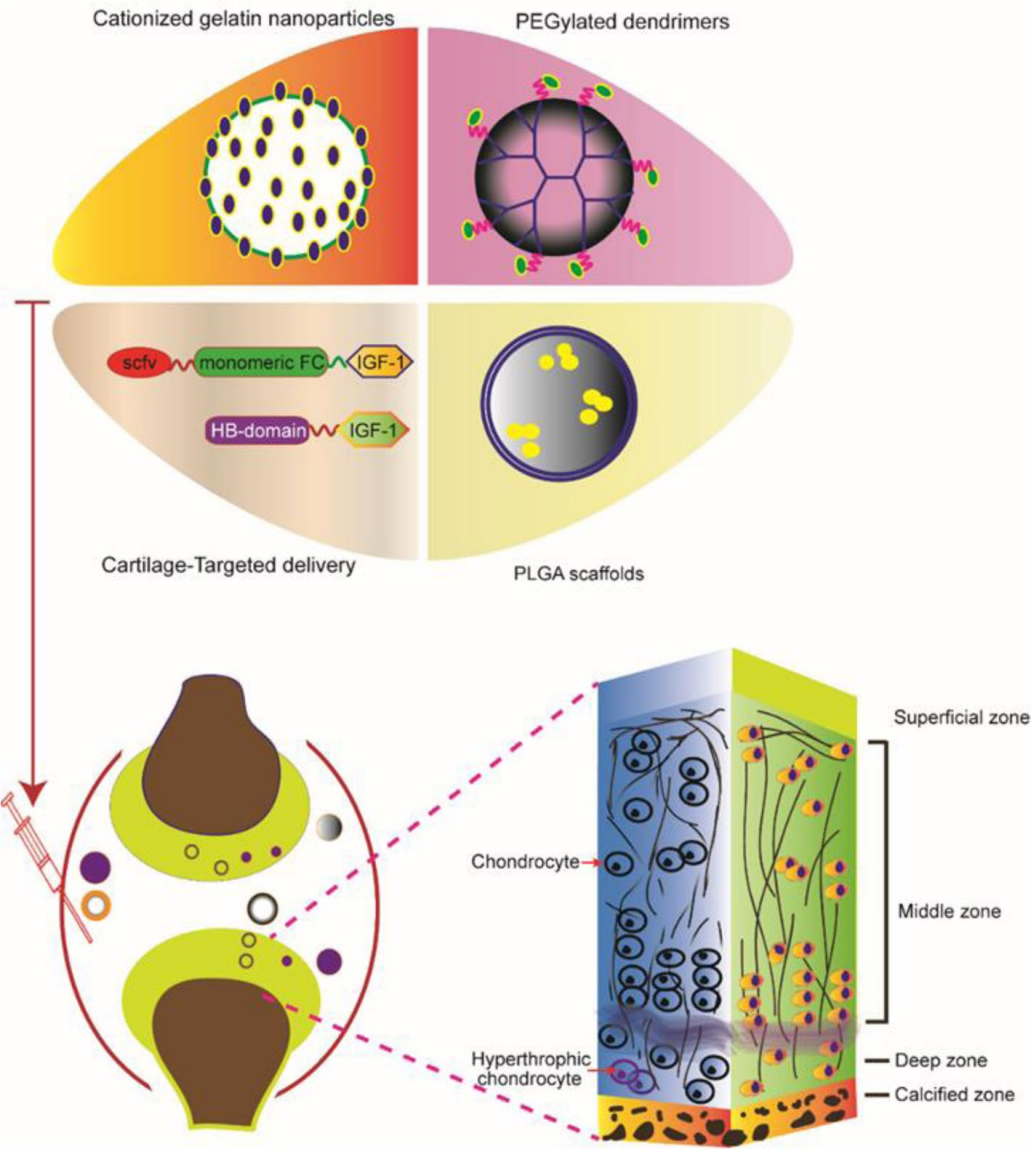
IGF-1 protein delivery for cartilage repair. Cationized gelation nanoparticles, PEGylated dendrimers IGF-1, PLGA, and fusion protein of IGF-1 with the HB domain of heparin-binding epidermal growth factor-like growth factor or scFv-Fc were used to target delivery of IGF-1 to reduce cartilage degeneration. Conjugating IGF-1 protein into the scaffolds can penetrate into thick cartilage

Steven Lu et al. [50] made a biodegradable hydrogel composite scaffold with macromonomer oligo (polyethylene glycol) fumarate (OPF), which simulated the structural layer of the osteochondral unit. IGF-1 and bone morphogenetic protein 2 (BMP-2) were loaded into gelatin particles. The study results showed that the double-layer OPF hydrogel complex had a robust spatial orientation for cartilage tissue repair and tremendous growth factor release potential. IGF-1 and BMP-2 synergistically promoted subchondral bone formation. Figure 3 shows that the delivery of IGF-1 protein to each layer of cartilage plays a therapeutic role.

Moreover, tissue or cell-specific delivery of the gene vectors by designed carriers enables an advanced version of gene therapy with reduced toxicity or risk [73]. In recent years, great progress has been made in engineered exosomes based on genetic modification. Exosome engineering using genetic and chemical methods for targeted drug delivery boasts low toxicity, low immunogenicity, and high engineerability, which holds promise for cell-free therapies [74]. Liang et al. [75] showed a genetic engineering strategy to modify exosome surfaces, achieve excellent chondrocyte-specific targeting and cartilage penetration, and rejuvenate the OA cartilage. Xu et al. [76] reported that targeted delivery of kartogenin (KGN) to synovial fluid MSCs (SF-MSCs) by engineered exosomes leads to even dispersion of KGN in the cytosol and strongly promotes the chondrogenesis of SF-MSCs in vitro and in vivo. These suggest that the delivery of IGF-1 through an engineered exosome system is a valuable research direction.

## Conclusion

IGF-1 profoundly regulates cartilage repair by promoting cartilage anabolism and inhibiting cartilage catabolism. Besides, IGF-1 may delay OA progression. Effective delivery of IGF-1 to injured cartilage is a prerequisite for growth factors to exert biological functions in vivo. Current IGF-1 delivery technologies based on viral or nonviral systems need further optimization. Delivery of IGF-1 through engineered exosomes may become an efficient way in the future. However, more in-depth studies should be performed to analyze IGF function and safety before its clinical application. In summary, with the advancement and optimization of delivery systems, IGF-1 will become an alternative cartilage repair strategy for OA treatment in the future.

## Data Availability

Not applicable.

## Abbreviations

ACI: Autologous chondrocyte implantation
Ad: Adenoviral
AAV: Adeno-associated virus
AD-MSCs: Adipose-derived mesenchymal stem cells
BM-MSCs: Bone marrow-derived MSCs
BMP: Bone morphogenic protein
CaAb: Cartilage-targeted single-chain human antibody fragment
ECM: Extracellular matrix
ERK: Extracellular signal-regulated kinase
HIF-1α: Hypoxia inducible factor-1α
IGF: Insulin-like growth factor
IGF1R: Insulin-like growth factor 1 receptor
IRS: Insulin receptor-substrate
IL: Interleukin
KGN: Kartogenin
MACI: Matrix-induced ACI
MAPK: Mitogen-activated protein kinase
MMP: Matrix metalloproteinases
MSCs: Mesenchymal stem cells
mTOR: Mammalian target of rapamycin
NF-κB: Nuclear factor kappa light chain enhancer of activated B cells
NPs: Nanoparticles
OA: Osteoarthritis
PI3K: Phosphatidylinositol 3′-kinase
SF: Synovial fluid
OPF: Oligo (polyethylene glycol) fumarate
TGF-β1: Transforming growth factor-β1
TNF-α: Tumor necrosis factor alpha
Xp-HB-IGF-1: Heparin bound to IGF-1
RA: Rheumatoid arthritis
FOXO: Forkhead-box class O
SA-β-gal: Senescence-associated β-galactosidase
